# A Bio-Inspired Methodology of Identifying Influential Nodes in Complex Networks

**DOI:** 10.1371/journal.pone.0066732

**Published:** 2013-06-14

**Authors:** Cai Gao, Xin Lan, Xiaoge Zhang, Yong Deng

**Affiliations:** 1 School of Computer and Information Science, Southwest University, Chongqing, China; 2 School of Engineering, Vanderbilt University, Nashville, Tennessee, United States of America; University of Maribor, Slovenia

## Abstract

How to identify influential nodes is a key issue in complex networks. The degree centrality is simple, but is incapable to reflect the global characteristics of networks. Betweenness centrality and closeness centrality do not consider the location of nodes in the networks, and semi-local centrality, leaderRank and pageRank approaches can be only applied in unweighted networks. In this paper, a bio-inspired centrality measure model is proposed, which combines the *Physarum centrality* with the *K-shell index* obtained by K-shell decomposition analysis, to identify influential nodes in weighted networks. Then, we use the *Susceptible-Infected* (SI) model to evaluate the performance. Examples and applications are given to demonstrate the adaptivity and efficiency of the proposed method. In addition, the results are compared with existing methods.

## Introduction

It is of theoretical significance and practical value to know how to identify influential nodes effectively in complex networks [1]–[14], such as controlling rumor and disease spreading [15], electric power supply [16], evolution of cooperation [9], [17]–[19], game theory [20]–[22], link prediction [23]–[25] and robust reproduction of organisms [26].Various centrality measures have been proposed over the years to capture network entities meanings 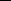 influence [27], importance [28]–[30], popularity [6], [31], controllability [32], [33] and spreading efficiency [34]. Three best known centrality measures were developed to distinguish which nodes are more central than others in binary networks, namely, *degree, closeness and betweenness centrality* [35]. Degree centrality based on counting the first neighbors of a node has an advantage of simplicity, ignoring the global structure. Closeness centrality was defined as the inverse sum of shortest distances from a focal node to all other nodes, which means high closeness centrality score is, a node more close to the others. However, if a node is at a dead-end, its removal will be without any effect in contrast with the case of a cut-vertex (the analog of a bridge for edges) which leads to disconnected components. Another important centrality is betweenness centrality, It is calculated by assessing the degree to which a node lies on the shortest path over the total number of shortest paths. These three centrality measures have already been extended to be applied in weighted networks. In 2011, a random-walk-based centrality called *LeaderRank* is proposed in [6], which can identify leaders in social networks better than the well-known *PageRank* algorithm [36], [37]. After that, Chen et al [27] developed a centrality method called *semi-local centrality* as a tradeoff between local degree centrality and other global but time-consuming measures with limitation to unweighted networks. Besides, there are also spectral centrality measures such as the *eigenvector centrality* [38], *alpha centrality* [38], *Katz's centrality* [39] and *subgraph centrality* [40].

However, in many real networks, edges are with with some form of attribute or weight. In addition, network can be changed dynamically. It is necessary to develop a new method to identify influential nodes with a adaptive manner. In 2012, an amoeboid centrality measure called *Physarum centrality* is proposed in [41], which can be used in weighted dynamic networks. *Physarum polycephalum*, as an amoeboid organism, can form a dynamic tubular network connecting discovered food sources. Furthermore, it has been applied on many fields, such as transportation [42], optimization [43], [44]. Concretely, Physarum can only control the flux through its body tube dynamically and then adapt itself to find optimal paths connecting two specified nodes conveniently, which means physarum centrality is likely to be applied in dynamically changed large networks suitably.

However, in contrast to common belief, there are plausible circumstances where the best spreaders do not correspond to the most highly connected or the most central nodes [34]. It has been proved that topology of networks plays an important role in spreading process. For example, if a hub (a node with high degree) exists at the end of a branch at the periphery of a network, it will have a minimal impact in the spreading process, whereas a less connected person who is strategically placed in the core of the network will have more influence on other individuals through a large network.

In this paper, a new method is proposed based on combining Physarum centrality and the layer of nodes located in networks. By using the *K-shell decomposition analysis* [45], [46], the *K-shell index* of nodes are obtained, which can be used to distinguish the relative location of a node in networks. Then we use the *Susceptible-Infected* (SI) model [47], [48] to evaluate the performance of the top-L nodes' spreading influence ranked by different centrality measures. Some existing centrality methods, such as semi-local centrality, physarum centrality, LeaderRank and PageRank approach, are used to compared with the proposed method.

The rest of the paper is organized as follows. Section 2 begins with a brief introduction to Physarum centrality. In Section 3 our method is proposed. Then, we use the SI model to evaluate the performance between previous approaches. The proposed method applied on simple numerical example and three real-world networks in Section 4. Finally, in Section 5, we give our conclusions.

## Basic Theories

### Physarum Model for Path Finding

A mathematical model for cases with two food sources has previously been proposed [49]. In brief, this model represents the shape of physarum cell body by a graph, in which an edge corresponds to a plasmodial tube and a node corresponds to a junction between tubes. At the beginning, there is an undirected weighted network which is strongly connected. Physarum can find the shortest path between starting node s and ending node t (node s and t correspond to food sources). Suppose that the variable 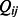 means the flux through the edge 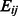 between nodes i and j, at which pressures are 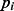 and 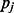, respectively. According to *Poiseuille flow*, the flux 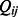 is denoted as,
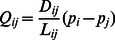
(1)where 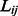 and 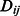 are the length and conductivity of the tube corresponding to the edge 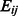, respectively. 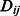 is its conductivity which is assigned with a value that belongs to (0,1] in the initialization.

At each node i (except the nodes s and t which are presented as two food sources), the total flow must be balanced as,
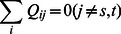
(2)Hence by considering the conservation law of flux we have,
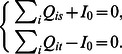
(3)where 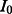 is the flux flowing into the starting node s and out of the ending node t, which is constant.

Then the network *Poisson equation* derived from Eqs. 1
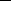
3 is as follows,
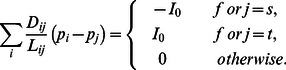
(4)


In order to show the positive feedback mechanism that a tube thickens depending on increasing flux and thins with decreasing flux, the conductivity 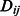 is assumed to change over time according to the flux 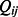,
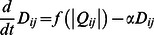
(5)where 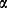 is a decay rate of the tube. 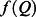 is a increasing function with 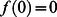. More description of 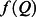 can be found in [49], [50]. The process above is just one iteration. The next is to judge whether the termination criterion is met or not. If the specified criterion is fulfilled, tubes without flux are cut off while others complete optimal paths. Meanwhile, update pressure at each node. The iteration will be stopped until the shortest path is found.

### Physarum Centrality

In weighted networks, the extension of degree centrality is defined as [51],>
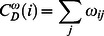
(6)


Inspired by it, physarum centrality of a node 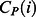 is defined as the sum of the criticality of each edge linked to it,
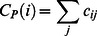
(7)where 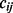 means the criticality of edge linked by node i and j. The value of 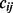 is calculated by using physarum model for path finding between all pairs of nodes in undirected weighted networks.

In the abovementioned model, physarum model can find optimal paths between any pair of nodes, by adapting the flux through each edge and its conductivity. When the adaptation is finished, optimal paths are reserved while other tubes fade away since no flux is passing though them.

In order to capture this characteristic, the criticality of the edge 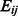 is defined as the sum of flux through it,
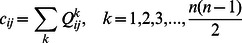
(8)where 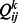 denotes the 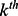 final flux through edge by using the physarum mathematical model, while different k implies different path finding between different pairs of food sources nodes s and t.

### Proposed Methods

Analyzing the definition of physarum centrality, it seems that physarum centrality is defined as a tradeoff between the extension of degree centrality and betweenness centrality. Physarum finds the shortest path with flux passing through tubes. 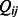 is the amount of flux on the edges. Then a node's centrality is the sum flux of the edges linked to it. It has been shown that physarum has advantages of flexible self-adaptability and less computational time than Dijkstra's algorithm [52]. Inspired by this, physarum may show a superiority for the adaptive dynamics of networks, in the cases of traffic congestion or following accidents. Therefore, physarum will quickly adapt itself to identify newly influential nodes, when some randomly selected nodes of top-L ones in a network are removed.

Just like degree centrality and betweenness centrality, physarum centrality only captures the characteristics in the aspects of degree, shortest path, rather than location of the network. In contrast to common belief, it seems to be more possible that the most efficient spreaders are those located within the core of a network, rather than highly connected or the most central ones on the edge location [34]. Here we use the k-shell decomposition analysis to identify the location of a node in the network. By using this well-established tool, each node will be assigned with k-shell index value, 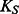, to each node, representing its location in the network. If the 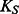 value of a node equals to 1, it means that the node is located on the periphery of the network.

**Figure 1:**
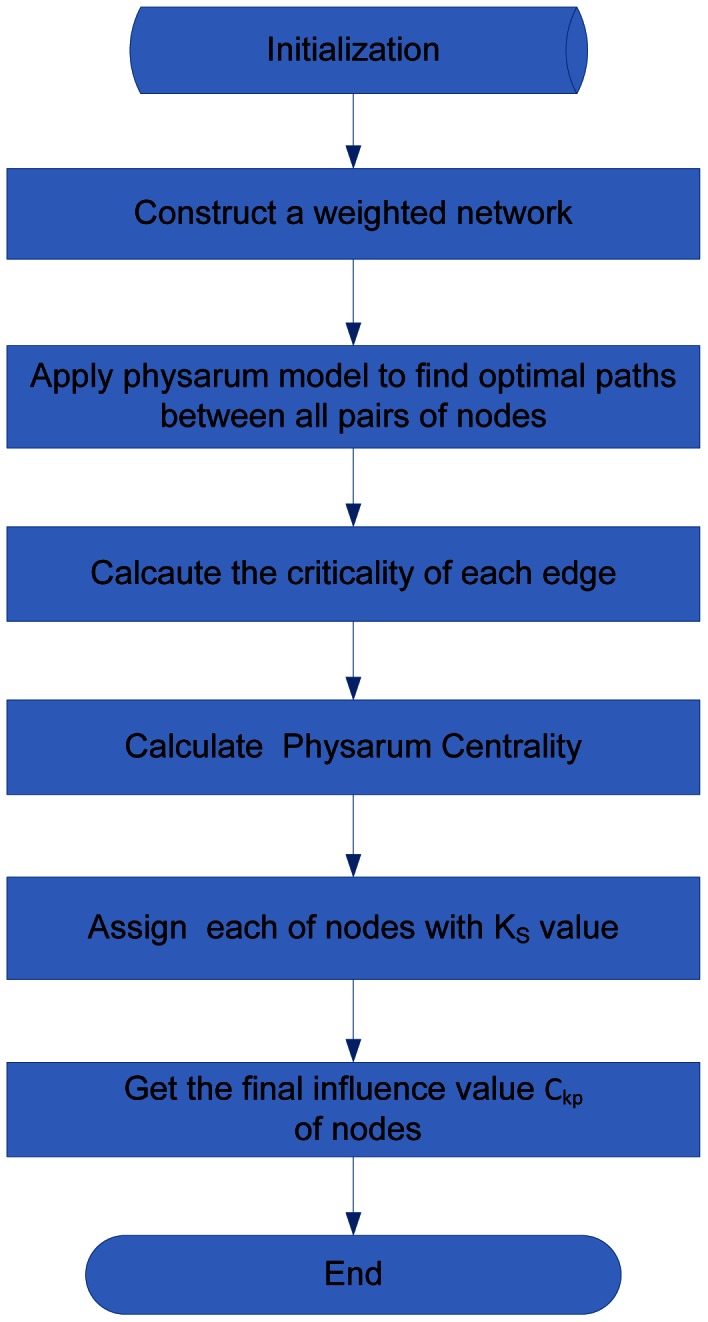
The flow chart of the proposed method.

In our approach, two factors of a node 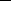 physarum centrality and k-shell index in the network, are both taken into consideration. The flow chart of the proposed method is shown in Figure 1.

Step 1. Construct a undirected weighted network. Since weights in most weighted networks stands for tie strength, rather than the length between two individuals. the edge weights need to be reversed, in order to correspond to the tube length in physarum model.

Step 2. Apply physarum model to find optimal paths between all pairs of nodes.

1. The conductivity of each tube 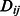 is assigned with 0.5.

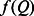
 in Eq. 5 adopts 
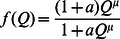
 with parameters set a  = 2, 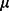 = 27.
3. Termination criterion is is determined by maximum iterations, which is 4logn.

Step 3. Calculate the criticality of each edge by Eq. 8 with recorded values 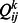.

Step 4. Calculate physarum centrality of nodes with Eq. 7.

Step 5. By using the tool of k-shell decomposition analysis, each node will be assigned with 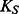 value. According to its decomposition process, first of all, nodes of degree one have 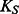 index equal to one. Then prune all these nodes and the links incident on nodes with one connection from the network. Nodes that have degree one on the reduced graph are assigned 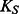 index of one and recursively pruned. Secondly, the same is done for nodes with two connections and so on, until all nodes are pruned from the network. Lastly, normalization is necessary.
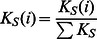



Then, the final influence value of nodes ranked by the proposed method, 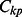
(short for K-shell Physarum Centrality) is expressed as follows,
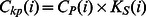



Hence, nodes located in the core of networks have larger 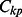 value than ones in the periphery of a network.

### Illustrative Examples

In this section, two simple examples and two applications in real networks are used to evaluate the performance of centrality measures. Here, a comparison with another several centrality measures (degree, closeness, betweenness and physarum in weighted networks and semi-local centrality, LeaderRank and PageRank in unweighted networks) is also provided to shown the differences among them.

### Two numerical Examples

**Figure 2:**
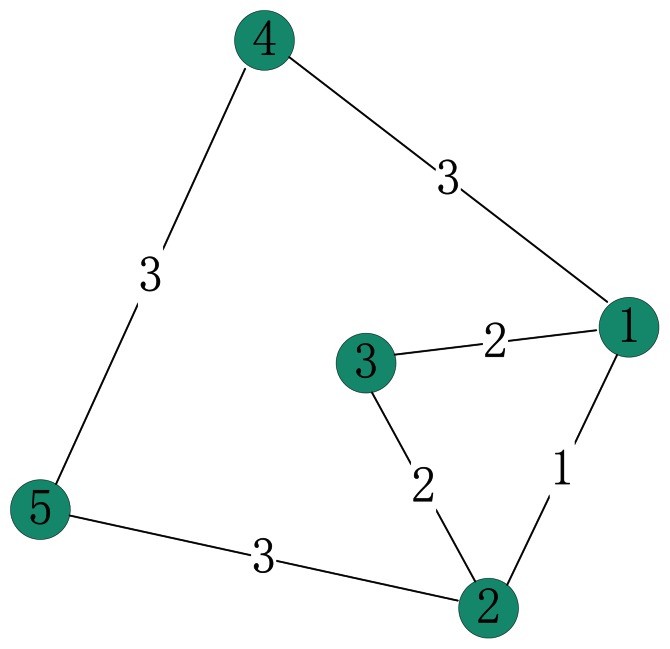
A weighted example network.

The first simple example is a weighted network with 5 nodes and 6 weighted edges, which is adopted from [53], as illustrated in Figure 2.

Due to symmetry of the network, the influence scores of node 1 and 2, or node 4 and 5 should be the same, regardless of which centrality measure is taken. However, the results listed in Table 1 show that degree centrality ranks the node 1, 2, 4 and 5 as the same ranking score, while betweenness centrality, closeness centrality and our method have consistent results as node 4 and 5 have greater centrality value than node 1 and 2.

**Table 1:** Influence scores based on different centrality methods for network in Figure 2.

v	K(v)	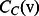	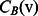	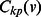
1	6	0.1714	1	0.2086
2	6	0.1714	1	0.2086
3	4	0.1607	0	0.1601
4	6	0.1978	2	0.2113
5	6	0.1978	2	0.2113

**Figure 3:**
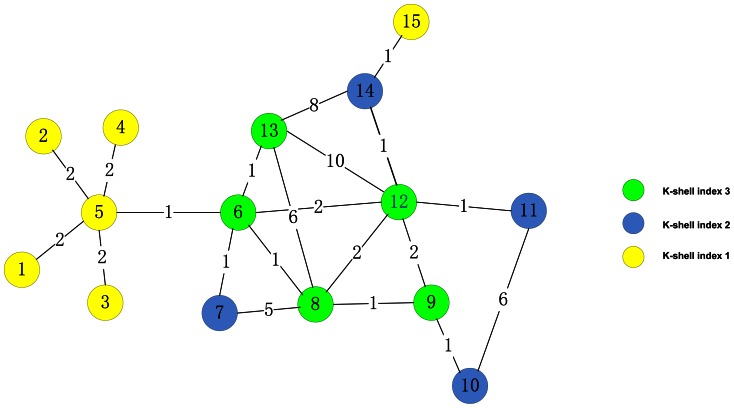
A weighted example network with 15 nodes and 21 weighted edges. K-shell decomposition analysis is applied to this network. Different 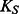 value paint in different colors. Nodes 1, 2, 3, 4, 5 and 15 colored in yellow are at the periphery of the network. Nodes 7, 10, 11 and 14 colored in blue are located at the second layer. Nodes 6, 8, 9, 12 and 13 are apparently on the core of the network.

To further illustrate the difference between our method and other centrality measures in weighted and unweighted networks, respectively, we develop another example from [54]. As illustrated in Figure 3, node 1, 2, 3, 4, 5 and 15 colored in yellow are located at the periphery of the network and assigned with 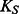 value 1. Node 7, 10, 11 and 14 with 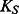 value 2 are on the second layer. Node 6, 8, 9, 12 and 13 are at the core of the network.

To evaluate the performance, we use a variant of the SI model adopted from [55] to study the dynamical evolution of epidemic spreading process in weighted networks. In this model, individuals can be in two discrete states: (i) Susceptible S(t) represents the number of individuals susceptible to the disease, not yet infected; (ii) Infected I(t) denotes the number of individuals that have been infected and are able to spread the disease to susceptible neighbors. At each step, one node is set to be infected initially and Then each infected node spreads disease or information to randomly one of its susceptible neighbors with probability 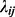 in weighted networks (such a model is usually to mimic the limited spreading capability of individuals),
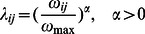
(9)


where 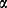 is a positive constant and 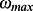 is the largest value of 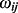 in the network. For weighted networks, we assume that weight 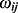 denote connection strength through link 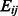. For example, more familiar two individuals (with larger weight) may infect each with greater probability. Since 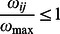, the smaller 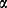 is, more quickly the disease or information spreads. Here we use the total number of infected nodes at time t, denoted by F(t) as an indicator of influence evaluation. Larger F(t) value of a node is, larger spreading ability the node has. The process stops when there is no susceptible node to be infected, namely, at a stable state, denoted by F(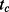).

According to the results in Table 2, regardless of unweighted network or weighted network, node 6 have a larger F(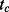) value than node 5 due to its core position in the network, but physarum centrality (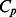) ranks node 5 higher than node 6. After taking the k-shell index into consideration, node 6 ranked by the proposed method is more central than node 5, which is consistent with ranking order by the SI spreading model. In the weighted network, the top-1 ranked by the proposed method is node 12 which has the largest F(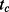) (larger than node 6 ranked by closeness centrality and betweenness centrality as the top-1 node, node 13 by degree centrality). In addition, all the nodes with 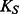 value 3 have larger spreading ability than others. This demonstrates the k-shell index of nodes plays an important role of ranking influential nodes in networks.

**Table 2:** Simulations of effectiveness on the network illustrated in Figure 3.

		Unweighted network					Weighted network					
*v*	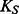 (*v*)	LR(*v*)	PR(*v*)	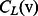	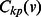	F( 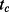 )	K(*v*)	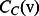	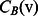	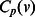	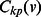	F( 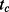 )
1	1	0.625	0.356	32	0.011	4.81	2	0.016	0	0.021	0.009	4.39
2	1	0.625	0.356	32	0.011	4.98	2	0.016	0	0.021	0.009	5.47
3	1	0.625	0.356	32	0.011	4.74	2	0.016	0	0.021	0.009	4.77
4	1	0.625	0.356	32	0.011	5.14	2	0.016	0	0.021	0.009	4.10
gray!255	1	1.458	1.423	79	0.084	5.34	9	0.026	92	0.160	0.071	7.38
gray!256	3	1.458	0.978	188	0.265	7.94	6	0.030	120	0.157	0.209	7.76
7	2	0.833	0.432	88	0.024	9.61	6	0.022	0	0.025	0.022	6.76
8	3	1.458	0.923	177	0.113	10.01	15	0.026	32	0.067	0.089	8.10
9	2	1.042	0.617	112	0.052	8.01	4	0.022	26	0.048	0.043	8.00
10	2	0.833	0.480	37	0.031	8.10	7	0.017	0	0.035	0.031	6.31
11	2	0.833	0.469	67	0.038	7.83	7	0.018	0	0.043	0.038	6.91
12	3	1.667	1.117	185	0.182	9.99	18	0.024	70	0.162	0.215	8.65
13	3	1.250	0.762	162	0.093	10.51	25	0.022	0	0.137	0.182	7.88
14	2	1.042	0.676	97	0.064	9.77	10	0.019	26	0.060	0.054	7.37
15	1	0.625	0.308	22	0.011	6.46	1	0.015	0	0.021	0.009	3.90

The corresponding unweighted network of Figure 3 is obtained by letting the weight of all edges be 1. Initially, only one node is infected with(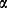 = 1). For each initial node, F(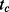) is obtained by averaging over 100 steps.

### Applications in Real-world Networks

In this section, applications to three real networks are given to demonstrate our the flexible adaptability and efficiency of the proposed method. (i) The US Air line network 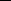 it has 322 air ports and the air line between two air port can be denoted as a connection between two nodes in the network. The data can be downloaded from 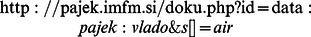. (ii) Club network [56] 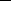 the undirected Zachary's “karate club ” networks of 1977. The data are collected from the members of a university karate club by Wayne Zachary over two years. Zachary constructed a weighted network by denoting each member in the club as a node. Each edge in the network represents the connected two members are friends outside the club activities and its weight indicates the relative strength of the associations (number of situations in and outside the club in which interactions occurred). (iii) Citation network [57] 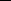 this data set consists of paper and citation relationship chosen from Arnetminer. There are 235 nodes and 411 edges in this unweighted network. The basic topological properties of these three networks are shown in Table 3.

**Table 3:** The basic topological features of the two real networks.

Network	n	m	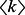	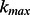	C	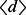
Club	34	231	13.59	48	0.2557	2.4082
Air lines	332	2126	12.81	139	0.3964	2.7381
Citation	235	411	1.75	35	0.0994	3.1494

n and m are the total numbers of nodes and links, respectively. 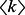 and 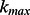 denote the average and the maximum degree, respectively. C is the clustering coefficient. 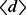 is the average shortest distance.

In the US Air lines network, the initial ranking of top-20 air ports by the proposed method are listed on the 2nd column in Table 4. Then, three air ports of the top-10 are randomly selected to be removed 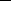 node 118, 261 and 313. In our common belief, after topology is changed, the ranking orders will not change at all. Actually, node 8 is ranked as second order at the initial state. After randomly removing three nodes, node 8 drops to the 10th place, rather than top 1. In this case, Dijkstra's algorithm needs to traverse the whole nodes network again, which leads to high computational complexity, while physarum can just adapt the flux though tubes dynamically and finally new influence score of each node will be obtained. The adaptivity of the proposed method is very useful to identify the influential node when the topology of networks is changed dynamically.

**Table 4:** The different order of top-20 nodes at the initial status and final status.

Top-20 nodes	Initial Ranking	Final Ranking
1	118	201
2	8	47
3	261	182
4	201	248
5	47	152
6	182	255
7	313	167
8	13	166
9	152	230
10	67	8
11	255	112
12	230	144
13	144	258
14	65	147
15	166	293
16	148	109
17	112	65
18	258	162
19	329	311
20	293	150

**Figure 4:**
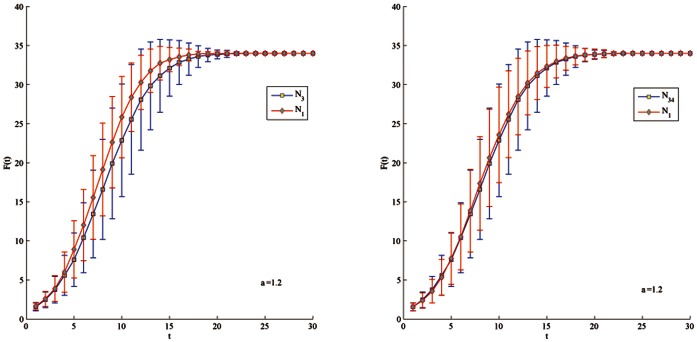
Comparison of spreading ability among node 1, 3 and 34. For each node, F(t) is obtained by averaging over 100 implementations (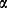 = 1.2).

**Figure 5:**
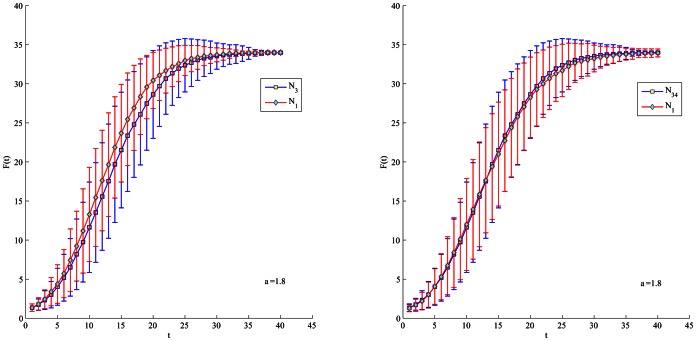
Comparison of spreading ability among node 1, 3 and 34. For each node, F(t) is obtained by averaging over 100 implementations (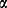 = 1.8).

**Figure 6:**
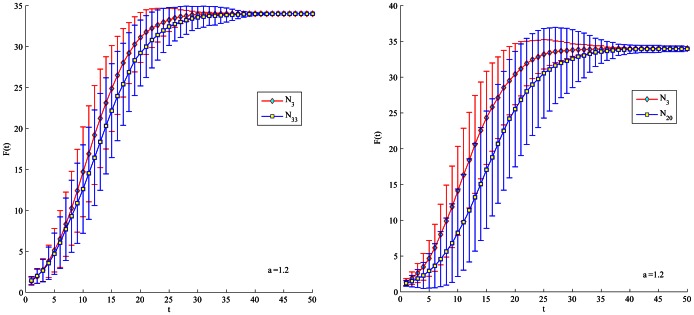
Comparison of spreading ability among node 3, 33 and 20. For each node, F(t) is obtained by averaging over 100 implementations (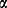 = 1.2).

**Figure 7:**
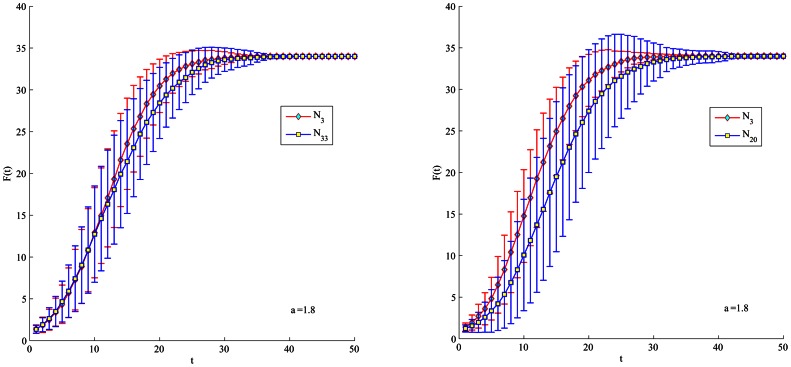
Comparison of spreading ability among node 3, 33 and 20. For each node, F(t) is obtained by averaging over 100 implementations (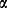 = 1.8).

In the Club network, the top-5 nodes ranked by the proposed method, degree centrality, closeness centrality and betweenness centrality are listed Table 5. Obviously, we need to distinguish the spreading ability among node 1, 3, 20 and 34 in order to efficiency of the proposed method. Here, we let infecting probability 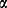 be 1.2 or 1.8 in order to slow down the spreading process. In Figures 4, 5, 6, 7, node 1 has greater F(t) value than node 3 regardless of 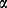
's value. There is subtle difference between node 1 and 34 in the aspects of spreading speed and stability. Besides, it is obvious that node 3 have much greater F(t) value than node 33 and 20 which is ranked by closeness and betweenness centrality as the third best influential node. Hence, the proposed method correctly identifies the most influential nodes in the Club network.

**Table 5:** The top-5 nodes ranked by the proposed method, degree, closeness and betweenness centrality.

Proposed method	Degree	Closeness	Betweenness
1	34	1	1
34	1	34	34
3	33	20	20
33	3	32	32
14	2	13	33

**Figure 8:**
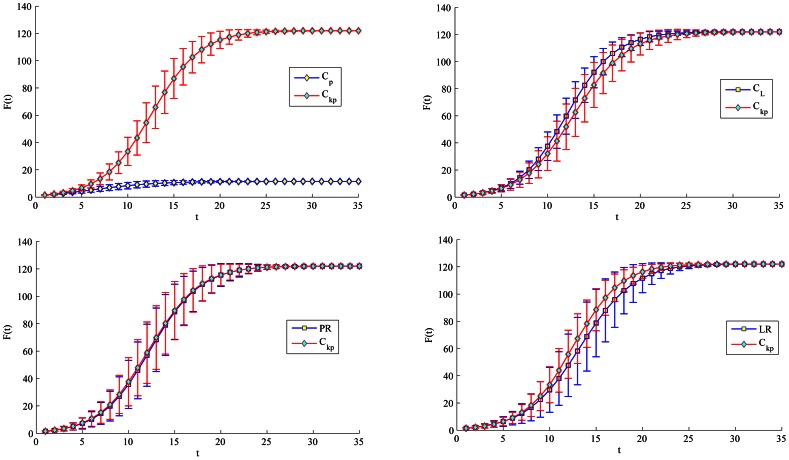
Simulation of top-5 nodes with initial infected to appear by our method with other four methods respectively (but not both). For each node, F(t) is obtained by averaging over 100 implementations (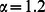).

**Figure 9:**
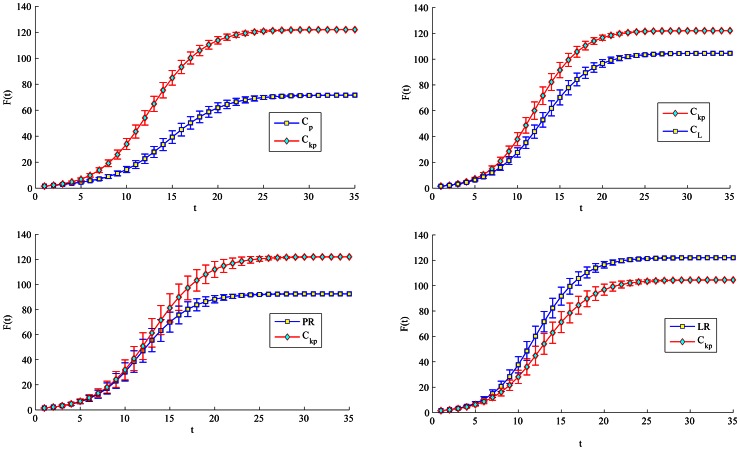
Simulation of top-20 nodes with initial infected to appear by our method with other four methods respectively (but not both). For each node, F(t) is obtained by averaging over 100 implementations (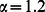).

**Figure 10:**
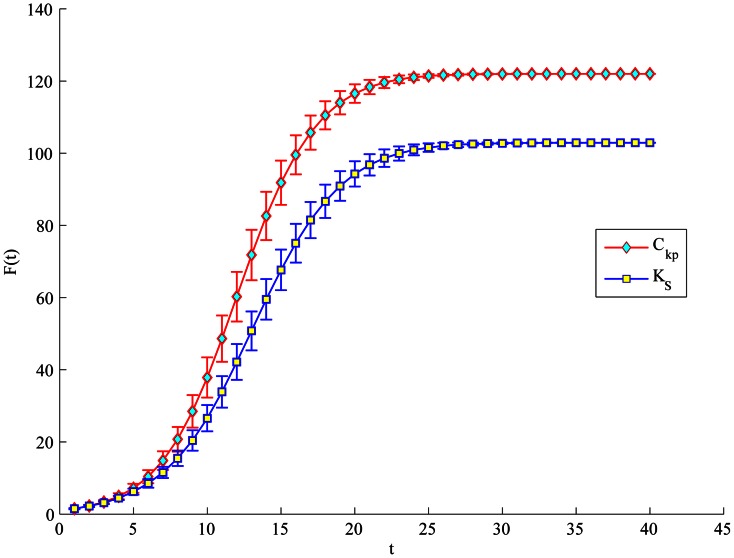
Simulation of top-20 with initial infected to appear by our method with k-shell index (but not both). For each node, F(t) is obtained by averaging over 100 implementations (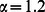).

Furthermore, when considering the Citation network, we set the initial infected to be the nodes either appear as the top-L (top 5 and top 20) by the proposed method or each of the four measures (such as physarum centrality, but not appear in both), as shown in Table 6. In Figure 8 and 9, no matter what the top-L nodes are, both show that the proposed method performs a quicker and wider spreading than purely physarum centrality. The top-5 ranked by semi-local centrality is little faster than the proposed method, but in turn, the top-20 nodes ranked by our method perform much better than semi-local centrality. Besides, LeaderRank can perform better than the proposed method when comparing top-20 nodes. However, it is notable that there is little difference between the proposed method, PageRank and LeaderRank in Figure 8. Furthermore, in Figure 10, due to the same 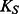 of the top-19 nodes ranked by purely K-shell index, there is no strictly top-5 in the condition. Therefore, we only compare the proposed method with purely K-shell index in the case of top-20. Obviously, top-20 nodes ranked by the proposed method have much more spreading ability than purely K-shell index.

**Table 6:** The top-20 nodes ranked by different methods.

Rank	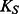 (*v*)	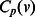	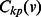	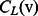	PR(*v*)	LR(*v*)
1	7(6)	3	7	32	7	32
2	8(6)	7	32	29	32	7
3	21(6)	175	29	7	29	29
4	23(6)	32	13	54	13	30
5	29(6)	29	53	23	30	53
6	30(6)	27	11	36	14	54
7	32(6)	50	30	69	53	14
8	33(6)	187	14	33	54	13
9	35(6)	22	58	53	98	36
10	36(6)	13	71	38	36	69
11	38(6)	63	3	30	58	38
12	44(6)	2	1	118	38	98
13	53(6)	11	98	87	69	33
14	54(6)	1	38	44	11	23
15	57(6)	136	87	57	27	87
16	69(6)	67	113	35	33	118
17	86(6)	82	72	21	87	58
18	87(6)	149	36	8	72	57
19	118(6)	150	54	86	3	72
20	12(5)	184	6	108	23	44

The top-19 nodes ranked by purely K-shell index are assigned with 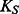 6.

## Conclusions

Identifying the most influential nodes in a weighted network has great physical and theoretical meanings. In this paper, a bio-inspired measure is proposed for identifying influential nodes in weighted networks. We have made a tradeoff between the physarum centrality and the k-shell index obtained by the k-shell decomposition analysis. To evaluate the performance, the SI model is used to distinguish the difference of top-L nodes ranked by different centrality measures. Compared with existing methods, experiment results show that the proposed method can well identify influential nodes, even in dynamic complex networks.
